# Can you estimate body composition in dogs from photographs?

**DOI:** 10.1186/s12917-016-0642-7

**Published:** 2016-01-20

**Authors:** Poppy Gant, Shelley L. Holden, Vincent Biourge, Alexander J. German

**Affiliations:** Department of Obesity and Endocrinology, University of Liverpool, Leahurst Campus, Chester High Road, Neston, Wirral CH64 7TE UK; Royal Canin Research Center, B.P.4-650 Avenue de la Petite Camargue, 30470 Aimargues, France; Present Address: Queen Mother Hospital for Animals, Royal Veterinary College, Hawkshead Lane, North Mymms, AL9 7TA UK

**Keywords:** Canine, Obesity, Body condition score, Adipose tissue

## Abstract

**Background:**

A validated method for assessing the visual characteristics of body condition from photographs (vBCS), would be a useful initial screening tool for client-owned dogs.

**Methods:**

In this retrospective study, photographs taken before and after weight loss from 155 overweight and obese dogs attending a weight management referral clinic were used in designing and testing the feasibility of vBCS. Observers with a range of experience examined the photographs, and estimated body condition indirectly (vBCS) using three different methods. In the first method (vBCS_measured_), the ratio of abdominal width to thoracic width (A:T) was measured, and cut-points used to determine body condition; the second method (iBCS_subjective_) involved semi-quantitative examination using visual descriptors of BCS; the third (vBCS_adjusted_) was a combined approach whereby A:T ratio was first determined, and the final score modified if necessary after assessing photographs.

**Results:**

When an experienced observer performed vBCS, there were moderate-to-good associations between body fat (measured by dual-energy X-ray absorptiometry) and the three vBCS methods (median R_s_: 0.51-0.75; *P* < 0.001), and also moderate-to-substantial agreement with actual BCS (median kappa 0.51–0.63; *P* < 0.001). For operators with a range of experience, moderate-to-substantial agreement was generally seen between actual BCS and the scores determined by all three methods (median Kappa 0.55–0.70, *P* < 0.001), but the strength of agreement varied amongst observers. Age, sex, breed, coat length, and coat colour had no significant effect on vBCS (*P* > 0.05 for all). Compared with ideal weight and obese dogs, errors in assessing body condition were more common for overweight dogs (e.g. BCS 6–7/9, *P* < 0.001) by vBCS_adjusted_ (*P* = 0.008), and vBCS_subjective_ (*P* = 0.021), but not by vBCS_measured_ (*P* = 0.150). For vBCS_adjusted_, body condition was most often overestimated whilst, for vBCS_subjective_, body condition was most often under-estimated.

**Conclusions:**

An estimate of body condition can be obtained from an indirect assessment of photographs, but performance varies amongst observers.

## Background

Obesity is one of the most common nutrition-related diseases observed in small animal practice [1], with studies indicating that between 34 and 59 % of dogs are overweight or obese [2–4]. Body condition scoring (BCS) is the best clinical method of assessing body composition [5, 6], with the 9-integer unit method being most widely accepted [5]. With this system, the observer assesses a range of characteristics both by palpation and by visual inspection, and then decides on a condition score category with reference to a series of silhouette images and descriptors. When BCS is performed by observers with training, there is good correlation with body fat mass determined by dual-energy X-ray absorptiometry (DEXA) [5, 6]; however, BCS is less reliable in the hands of inexperienced observers, especially owners who systematically under-estimate their dog’s body condition [7, 8].

Therefore, one limitation of conventional BCS is the need for it to be performed by an experienced observer after a hands-on assessment of the dog. This can be a problem both clinically, for instance if an owner were reluctant to present the dog at the veterinary clinic, and for large-scale epidemiological research studies where assessment by a single experienced observer would be impossible. If it were possible to design a system of body condition assessment that utilises visual characteristics alone, it might then be feasible to develop a tool for estimating body condition remotely. This would give owners reluctant to visit the veterinary clinic an easier option, and also provide a tool for large-scale epidemiological surveys conducted online. A study evaluating the use of photographs in assessing BCS have already been undertaken in dairy cattle, and the system performed relatively well [9]. However, an equivalent system of photographic assessment is not currently available for dogs. Therefore, the primary aim of the current study was to develop methods for assessing body condition indirectly (vBCS) from photographs, and to assess performance against body fat mass measured by DEXA and BCS determined by conventional means. A final aim was to determine the effect of a range of factors, including dog characteristics and observer experience, on the performance of vBCS methods developed.

## Methods

### Animals

Dogs were enrolled from referrals to the Royal Canin Weight Management Clinic (RCWMC), University of Liverpool UK, for investigation and management of obesity and associated disorders. The weight management protocol used by the clinic has been previously described in detail [10, 11]. To be eligible for inclusion, suitable photographs had to be available (see “photography and photographs” section below), taken either before or after weight loss. Owners of dogs had to have given permission for storage and use of the photographs for research purposes, and both body condition scoring (BCS) and body composition analysis had to have been conducted at the same time the photographs were taken. In addition, dogs had to be systemically well at the time of photography, and without significant abnormalities on complete blood count, serum biochemical analysis and urinalysis. The study protocol adhered to the University of Liverpool Animal Ethics Guidelines, and was approved by the University of Liverpool Research Ethics Committee (VREC50), the Royal Canin research Ethics Committee, and the WALTHAM Animal Welfare & Ethical Review Board. Owners of all participating animals gave informed written consent.

### Body composition analysis and body condition scoring (BCS)

Body composition was analysed with fan-beam DEXA (Lunar Prodigy Advance; GE Lunar), as previously described [10–12]. One observer (SLH) determined the actual BCS for each dog using a 9-integer unit BCS system [5]. This observer was experienced in performing BCS assessments, and her assessments were known to correlate strongly with body fat percentage determined by DEXA [6].

### Photography and photographs

**Fig. 1:**
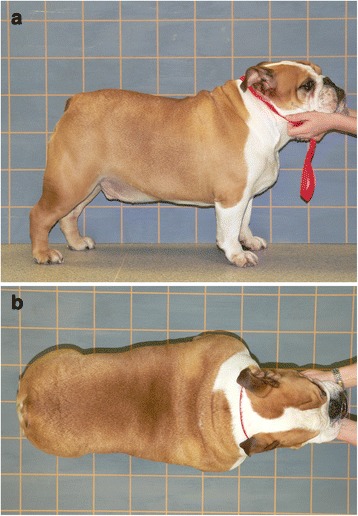
Photographic examples of the standardised views used for the study, taken in standing posture, from the side (**a**), and above (**b**)

**Fig. 2:**
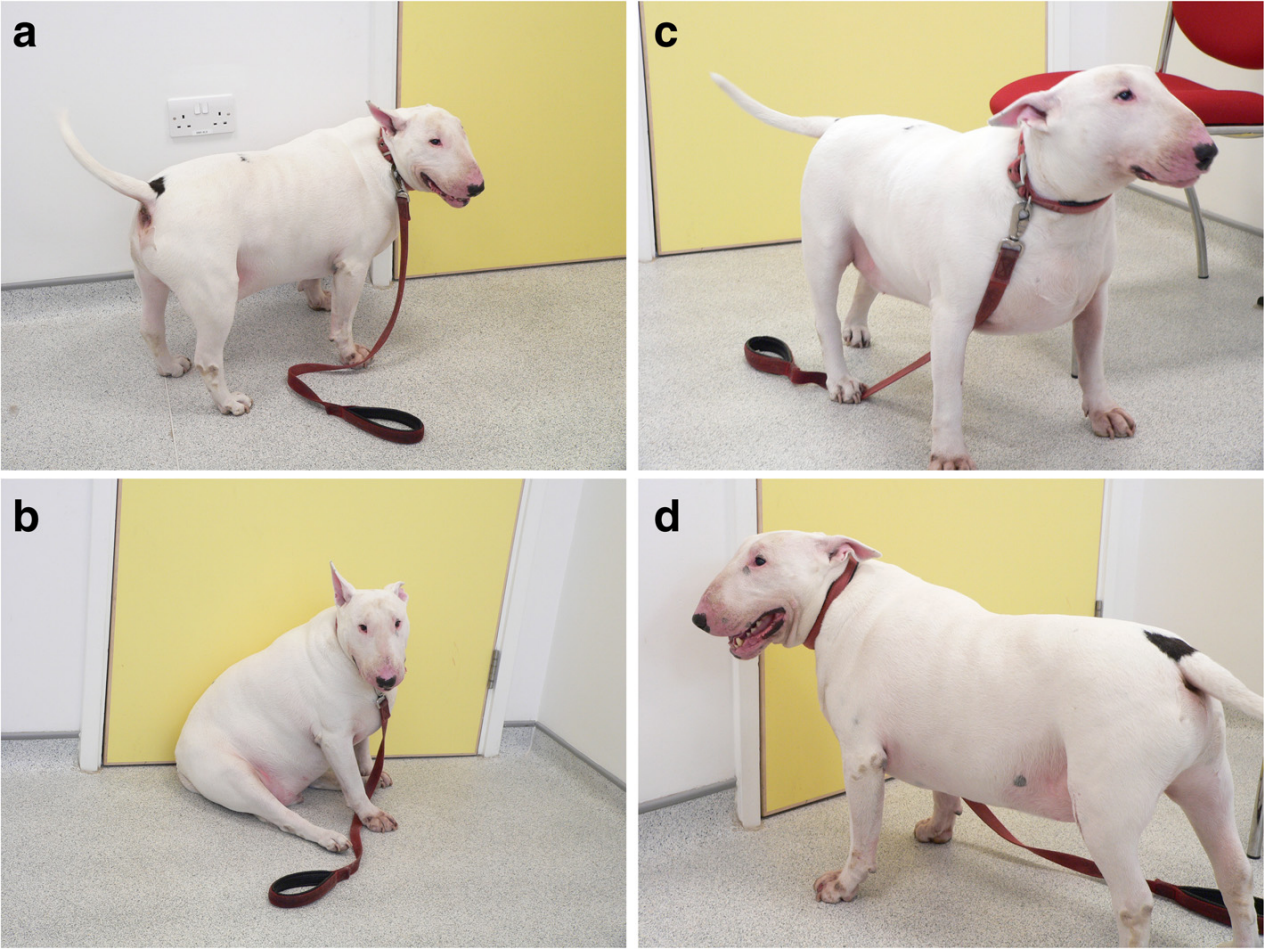
Examples of non-standardised photographs of the same dog taken from different views (**a** & **b**: side, **c**: front; **d**: behind) and in different postures (**a**, **c** & **d**: standing; **b**: sitting)

Two investigators (SLH and AJG) took all of the photographs, using a LUMIX DMC-FZ20 5 megapixel (2560 x 1920 pixel) digital camera with a LEICA DC-VARIO-ELMARIT 1:2.8/6–7 ASPH lens and a 12x optical zoom (Panasonic, Bracknell, UK). Photography was performed using the same approach both before and after weight loss. Some of the photographs were taken in a standardised manner, namely lateral and dorsal images taken against a grid with 100 mm x 100 mm markings (Fig. 1); other photographs were taken in a non-standardised manner, i.e. from various angles with the dog in a variety of postures (Fig. 2). The same protocol was used when taking all of the standardised photographs. For lateral photographs, the distance between the camera and the grid was approximately 3m, with the dog positioned immediately adjacent to the board and standing parallel to it. The photographer then adjusted their body position (e.g. by crouching or lying) to ensure the line of sight of the camera was perpendicular to the dog in the sagittal plane (i.e. perpendicular to the long axis of the body). For dorsal images, the distance between camera and grid was approximately 2m, with the dog standing directly on the grid such that its long axis was parallel to the grid lines. The camera was held directly above the dog, and its position adjusted so that the line of sight was perpendicular to the dog in the dorsal plane. All digital photographs were downloaded to computer and saved as Joint Photographic Experts Group (JPEG) files.

Between August 2012 and September 2012, one investigator (PG) reviewed the RCWMC Image Archive to select photographs for use in the study (Experienced Observer Set; Fig. 1; Tables 1 and 2). All of the photographs chosen were in focus, and taken in an adequately lit environment. A range of photographs were selected from dogs of different sexes, breeds, body size, coat colour and length, stage of weight management (i.e. both before and after weight loss), and body condition (e.g. BCS 4–9/9). A total of 125 standardised dorsal and lateral photograph pairs were selected (95 before weight loss; 30 after weight loss), taken from 101 of the dogs (Tables 1 and 2). A further 51 non-standardised photographs were used (37 before weight loss, 14 after weight loss), taken from 47 dogs (Fig. 1; Tables 1 and 2). The photographs were assigned a study number at random (using a list produced by random number generator; Stats Direct version 2.6.8; Stats Direct Ltd.), before being used in the studies. Forty-three of the dogs contributed photographs to both the standardised and non-standardised image set

**Table 1:** Breeds of dog used for generating the different sets of photographs used in the study

Experienced Observer Set				Multiple Observer Set			
Standardised		Non-standardised		Standardised		Non-standardised	
Pre-weight loss	Post-weight loss	Pre-weight loss	Post-weight loss	Pre-weight loss	Post-weight loss	Pre-weight loss	Post-weight loss
Akita	Border collie	Basset hound	Cairn terrier	Bichon frise	CKCS 3	Basset hound	CKCS 2
Basset hound	Cairn terrier	Border collie	CKCS 2	Bulldog 3	Corgi	Border collie	Cocker spaniel
BMD	CKCS 5	Bulldog	Cocker spaniel	Chihuahua	Crossbred 3	Bulldog	Crossbred 2
Bichon frise	Corgi	Bull terrier	Crossbred	CKCS	Doberman	Bull terrier	Doberman 2
Border collie 2	Crossbred 4	CKCS 5	Doberman 2	Crossbred 4	EBT	CKCS 2	Irish setter
Bulldog 3	Doberman	Crossbred 3	Irish setter	Dachshund	GR	Crossbred 2	JRT
Cairn terrier 3	EBT	Dachshund 2	JRT	Doberman	Irish setter	Dachshund 2	Labrador retriever 5
Chihuahua	GR 2	GR	Labrador retriever 6	EBT	JRT	Labrador retriever 8	Shipperke
CKCS 8	GSD	Labrador retriever	Shipperke	FCR	Labrador retriever 2	Lhasa apso	YT
Crossbred 13	Irish setter	10	YT	GSD		Poodle	
Dachshund 3	JRT	Lhasa apso		JRT		Pug 2	
Dalmatian	Labrador retriever 6	Poodle		Labrador retriever 5		Weimaraner	
Doberman 3	Pug 3	Pug 2		Lhasa apso		YT 2	
EBT	Shipperke	Weimaraner		Pug 2			
FCR	YT	YT 4		Shih tzu			
GR 5				YT 8			
GSD 2							
Irish setter							
JRT 3							
Labrador retriever 20							
Lancashire heeler							
Lhasa apso							
Poodle							
Pug 4							
Rottweiler							
Samoyed							
Shih tzu 2							
Shipperke							
Weimaraner							
YT 8							

*BMD* Bernese Mountain dog, *CKCS* cavalier King Charles spaniel, *EBT* English bull terrier, *FCR* flat-coat retriever, *F* female, *GR* golden retriever, *GSD* German Shepherd dog, *JRT* Jack Russell terrier

**Table 2:** Summary of signalment data for the dogs used for generating the different sets of photographs used in the study

Parameter	Experienced Observer Set				Multiple Observer Set			
	Standardised		Non-standardised		Standardised		Non-standardised	
	Pre-weight loss	Post-weight loss	Pre-weight loss	Post-weight loss	Pre-weight loss	Post-weight loss	Pre-weight loss	Post-weight loss
Age (mo)	72 (19–166)	86 (30–140)	84 (24–159)	85 (26–140)	77 (24–156)	84 (40–140)	84 (24–159)	85 (26–140)
Breed size	20 Toy	5 Toy	7 Toy	2 Toy	7 Toy	1 Toy	6 Toy	1 Toy
	16 small	7 small	7 small	5 small	4 small	4 small	3 small	5 small
	19 medium	7 medium	8 medium	1 medium	7 medium	5 medium	7 medium	1 medium
	40 large	11 large	12 large	9 large	7 large	5 large	9 large	8 large
Sex	M: 6, F: 5	NM: 17, NF: 13	M: 3, F: 1	F: 1, NM: 13, NF: 3	M: 1, F: 1	NM: 8, NF: 7	M: 2, F: 1	F: 1, NM: 12, NF: 2
	NM: 46, NF: 38		NM: 17, NF: 13		NM: 14, NF: 9		NM: 13, NF: 9	
Weight (kg)	31.5 (4.4–77.6)	16.6 (6.1–41.2)	22.7 (6.7–59.0)	32.2 (6.3–41.2)	20.9 (6.7–60.8)	25.8 (6.1–38.7)	23.5 (7.2–59.0)	32.2 (6.3–41.2)
Body fat (%)	45 (27–58)	30 (10–43)	43 (32–58)	31 (10–35)	43 (30–55)	31 (10–43)	43 (32–58)	32 (10–35)
BCS^1^	8 (6–9)	5 (5–6)	8 (6–9)	5 (5–6)	8 (6–9)	5 (5–6)	8 (6–9)	5 (5–6)
Coat length	Short 36	Short 11	Short 9	Short 4	Short 11	Short 6	Short 9	Short 4
	Medium 38	Medium 14	Medium 18	Medium 11	Medium 11	Medium 6	Medium 13	Medium 9
	Long 21	Long 5	Long 7	Long 2	Long 3	Long 3	Long 3	Long 2
Coat colour	Dark 39	Dark 10	Dark 10	Dark 10	Dark 8	Dark 5	Dark 7	Dark 9
	mid 13	mid 8	mid 8	mid 1	mid 7	mid 3	mid 7	mid 1
	mixed 8	mixed 8	mixed 5	mixed 4	mixed 5	mixed 4	mixed 5	mixed 3
	light 15	light 4	light 11	light 2	light 5	light 3	light 6	light 2

For continuous data and body condition score (BCS) results are expressed as median (range). For categorical data, the number of dogs in each category are listed. ^1^ BCS assessed with a 9-integer scale (7)

*M* male, *NF* neutered female, *NM* neutered male, *YT* Yorkshire terrier

**Fig. 3:**
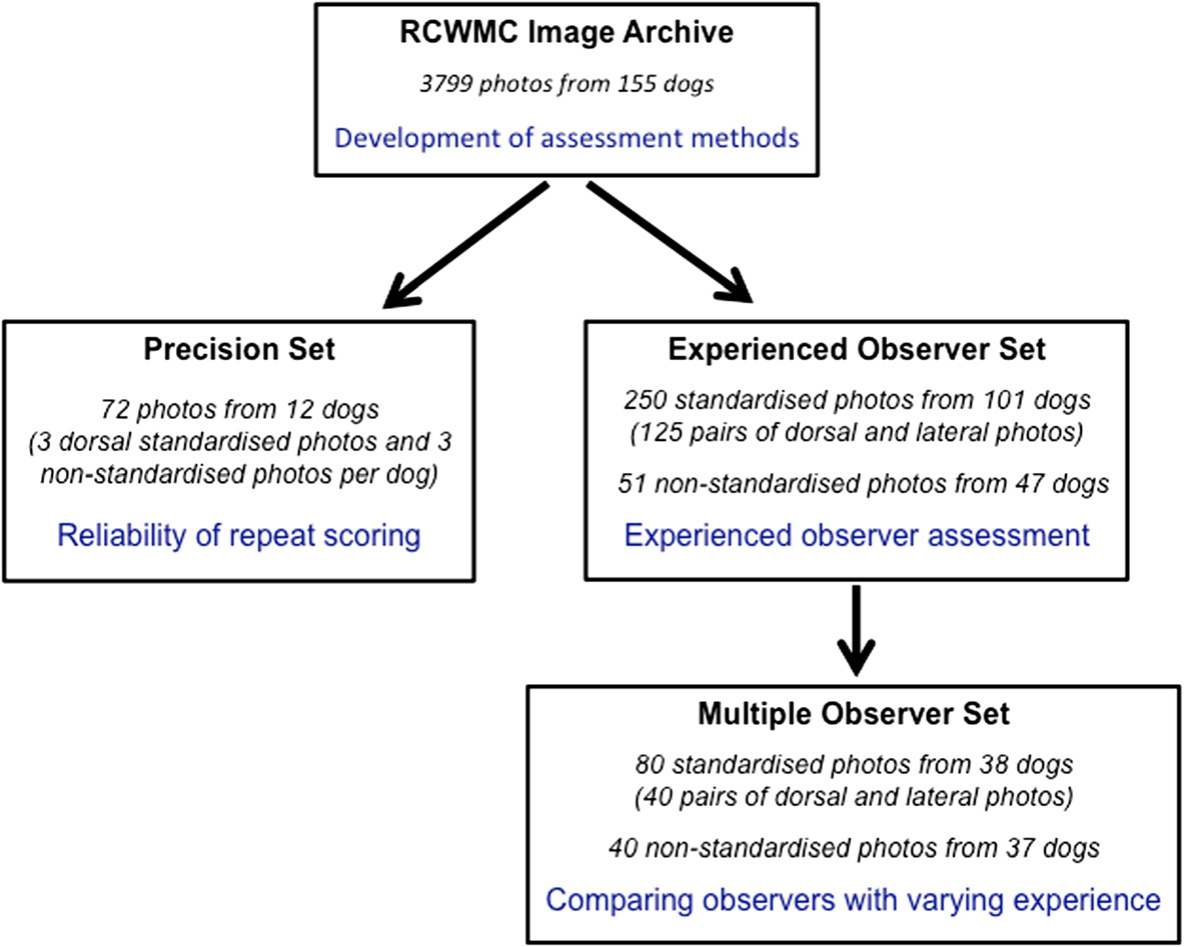
Flow diagram illustrating the make up of the image sets used for the different studies, and their relationship

For the multiple-observer studies, a subset of the photographs was used (Multiple Observer Set; Fig. 3; Tables 1 and 2), so as to provide a range of ages, breeds, sexes, coat types, and body condition scores. A total of 40 standardised photograph pairs and 40 non-standardised photographs were selected, all of which came from the Experienced Observer Set before any investigations commenced. Twenty-one of the dogs contributed photographs to both the standardised and non-standardised image set.

A second investigator (AJG) also selected a separate set of 72 photographs (Precision Set, Fig. 3) from the RCWMC Image Archive, for use in studies to determine the reliability of repeat vBCS assessments. Photographs from 12 dogs were included, with 3 dorsal and 3 non-standardised photographs from each dog. Again, the investigator ensured that they represented a range of breeds (corgi, Cavalier King Charles spaniel [CKCS], crossbred, JRT, Labrador retriever [4], Lhasa apso, and Yorkshire terrier [2]), sexes (8 neutered male, 4 female), BCS scores (median 7, range 5–9), coat lengths (3 short, 6 medium, 3 long), coat colours (3 dark, 1 mid, 4 mixed, 4 light), and stages of weight loss (7 pre-weight-loss, 5 post-weight loss). Dorsal and non-standardised photographs were separately coded using the random number generator function of a computer software programme (Stats Direct version 2.6.8),

### Development of methods for indirect body condition scoring

Prior to all validation studies, two observers (PG and AJG) subjectively examined a range of the photographs from the RCWMC Image Archive (Fig. 3) to decide upon possible objective (i.e. using measurements) and subjective methods for assessing body composition. From this, three methods of indirect body condition scoring (vBCS) were devised. The first was an objective method (vBCS_measured_) based upon the fact that the presence of a ‘waist’ is an important characteristic in conventional BCS systems [5]. In this method, the ratio of abdominal width to thoracic width (A:T ratio) was measured on a standardised dorsal photograph (Fig. 1). First, each JPEG file was opened and enlarged to occupy the whole screen of the monitor. Next, the width of the thorax at the widest point, and width of abdomen at the narrowest point were measured by using a ruler. Results were recorded (in millimetres) in a computer spreadsheet (Excel® for Mac 2011, Microsoft), and the ratio of A:T calculated using the function tool. A body condition score category was then automatically assigned based upon the A:T ratio (e.g. ideal weight, A:T < 0.77; overweight, A:T = 0.77–0.87; obese, A:T > 0.87). These cut-points were determined subjectively, to optimise the number of dogs correctly assigned (based upon their BCS and body fat percentage).

Second, a subjective method (vBCS_subjective_) was tested, whereby the observer examined a non-standardised photograph, and estimated body condition semi-qualitatively based upon visual descriptors of BCS [5]. Dogs were scored as either ideal weight, overweight, or obese, using an amalgamation of the characteristics of BCS 4/9 and 5/9, BCS 6/9 and 7/9, and BCS 8/9 and 9/9, respectively. Visual characteristics for the ideal weight category included readily observing a ‘waist’ from above, or an abdominal ‘tuck’ from the side. In addition, adipose tissue deposits were not evident in the neck, over the thorax, over the hips, nor around the tail base. Visual characteristics for the overweight category included an absent or barely discernable waist (when viewed from above) or abdominal tuck (when viewed from the side). In addition a covering of adipose tissue could be identified in the neck region, over the thorax, over the lumbar area and around the tail base. For the obese category, the waist (viewed from above) and/or abdominal tuck (viewed from the side) had to be absent, and abdominal distension had to be evident. In addition, marked adipose tissue deposition was evident in the neck region, over the whole of the thorax, in the lumbar area, over the hips and around the tail base.

The third method (vBCS_adjusted_) was a refinement of method 1, whereby the A:T ratio was first measured from a standardised dorsal photograph, and categorised into a suggested body condition score category. The observer could then modify the chosen score, if they wished, after subjectively assessing standardised dorsal and lateral photographs of the same dog. Visual BCS descriptors were again used, as for method 2.

### Precision of vBCS methods

One observer (PG) assessed all 72 photographs of the precision set on a single occasion. This person was blinded to the results of conventional BCS assessment and measurements of body fat by DEXA. For dorsal images, the A:T ratio was measured, and precision determined by calculating coefficients of variation (CV). For non-standardised photographs, the vBCS_subjective_ method was used and dogs were assigned to 1 of 3 categories (as above). Given that it would not have been appropriate to calculate CV for such categorical data, agreement amongst repeats was instead assessed using Kappa analysis.

### Experienced observer assessment of vBCS methods

One observer (AJG), with extensive prior experience of BCS assessment and body composition analysis in dogs, first measured A:T ratio on images from the single observer set. This person was also blinded to the results of conventional BCS assessment and measurements of body fat by DEXA. The associations between A:T ratio and body fat percentage, and also the influence of various factors was then assessed (see “statistical analysis” section below). The A:T ratio results were then used in the vBCS_measured_ assessment, and the same observer also performed the vBCS assessments. Comparisons were made between the results of all vBCS assessments and both body fat percentage and actual BCS (see “statistical analysis” section below). In addition, for each method, the proportion of correct BCS assessments was calculated, i.e. when the body condition category assigned by the vBCS method was equivalent to the actual BCS method. The proportion of incorrect BCS assessments was also determined along with the direction of the error, i.e. under-estimation and over-estimation compared with the actual BCS. Under-estimation was deemed to have occurred when the body condition category assigned by the vBCS method was less than that determined by the actual BCS result; an over-estimation was deemed to have occurred when the body condition category assigned by the vBCS method was greater than that determined by the actual BCS result.

### Comparison of vBCS performed by observers with varying experience

Twelve observers with a range of experience participated. Three of the observers were study authors including a veterinary surgeon (AJG), a veterinary nurse (SLH), and an undergraduate veterinary student (PG). The remaining 9 observers were known to the authors and participated after giving their informed consent in writing. Of these, 1 was a veterinary surgeon, 1 was a veterinary nurse, 2 were undergraduate veterinary students, and the remaining 5 people had no veterinary training. All observers were blinded to the results of conventional BCS assessment and measurements of body fat by DEXA. Given that the photographs used were drawn from the Experienced Operator Set, one observer (AJG) had already scored these images. So as to avoid repetition, the original assessments of this observer were carried forward to this study. The other observers examined all photographs in the multi-observer set in a single sitting, and estimated body condition (vBCS) using the three methods outlined above. To minimise any possible influence of the order of assessment, half of the observers examined the non-standardised images first and estimated body condition using the vBCS_subjective_ method; they then examined the standardised images and estimated condition using the vBCS_measured_, before completing the assessment by performing vBCS_adjusted_ using the standardised images. The other observers performed assessments in the opposite order, i.e. first performing vBCS_measured_ and vBCS_adjusted_ on the standardised images before performing vBCS_subjective_ on the non-standardised images.

As with the results of the single-observer studies, all vBCS scores were compared with body fat measurements and conventional body BCS (see “statistical analysis” section below). In addition, the proportions of correct and incorrect BCS assignments per observer were calculated for each method.

### Statistical analysis

Study data are available in the supplemental material (see Additional file 1). Data are expressed as median (range) except where indicated. Statistical analyses were performed with computer software (Stats Direct version 2.6.8), and the level of significance was set at *P* < 0.05 for two-sided analyses. For continuous data (e.g. age, A:T ratio and body fat percentage), the Shapiro-Wilk test was first used to assess whether or not data were normally distributed, and parametric and non-parametric tests were then used as appropriate. The association between A:T ratio and body fat mass was determined with Spearman’s rank correlation, whilst grouped linear regression was used to determine the effect of various factors (e.g. breed group, sex, coat length, coat colour) on this association. Non-parametric methods were exclusively used for both categorical data and data expressed as proportions. In this respect, Spearman’s rank correlation was used to determine the association between BCS and body fat percentage, whilst agreement was assessed using kappa analysis (when there was no ordering amongst categories) and weighted kappa analysis (when agreement across 3 categories was assessed e.g. body condition score categories). The level of agreement was judged subjectively based upon previously published criteria [13]. Briefly, results between 0.01 and 0.20 were classed as slight agreement; those between 0.21 and 0.40 were classed as fair agreement; those between 0.41 and 0.60 were classed as moderate agreement; those between 0.61 and 0.80 were classed as substantial agreement; and those between 0.81 and 0.99 were classed as almost perfect agreement. Finally, Fisher’s exact test was used to compare proportions of correct answers from the different vBCS systems and conventional BCS.

## Results

### Study animals

In total, 155 dogs were seen between February 2005 and August 2012. Of these, 105 dogs had suitable images for inclusion in the study. A range of ages, breeds, sexes, and coat types were included, and full details are given in Tables 1 and 2. Median body weight for all dogs was 26.3 kg (range 4.4–77.6 kg), median body fat was 43 % (10–58 %), and median BCS was 8/9 (5/9–9/9).

### Precision of vBCS methods

The median CV of repeat A:T ratio measurements, from the 12 dogs, was 2.4 % (range 1.4–10.2 %). When using vBCS_subjective_ on non-standardised images, there was a highly significant moderate agreement amongst repeat images of the same dogs (Kappa 0.41, *P* < 0.001).

### Experienced observer validation of vBCS methods

**Fig. 4:**
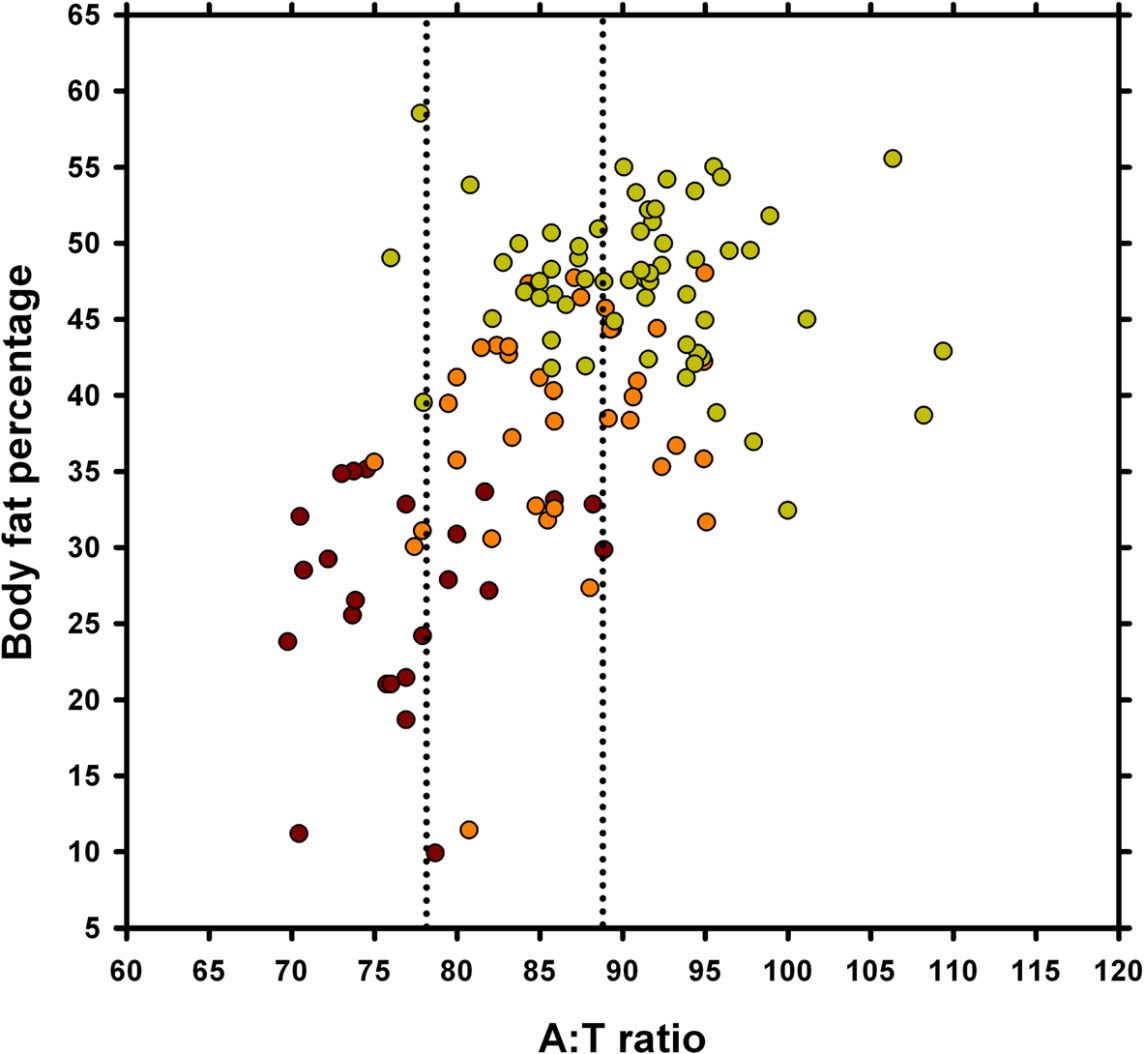
Association between abdominal:thoracic ratio and body fat mass measured by dual-energy X-ray absorptiometry. A moderate positive association with both body fat mass was seen (R_s_ = 0.50, *P* < 0.001)

When A:T ratio was measured by an experienced observer (AJG), a positive association was seen with body fat percentage measured by DEXA (R_s_ = 0.50, *P* < 0.001; Fig. 4), but this association was weaker than between conventional BCS and body fat percentage (R_s_ = 0.78, *P* < 0.001). Using grouped linear regression, none of the factors assessed (e.g. breed, sex, coat length, coat colour) had any effect on either the slope (*P* > 0.3 for all) or line separation (*P* > 0.2, for all).

The same observer (AJG) then determined body condition using the three vBCS methods (Table 3). The results from all three methods were positively associated with body fat percentage (*P* < 0.001 for all), and Kappa analysis revealed moderate-to-substantial agreement between actual BCS and body condition determined by all three methods (*P* < 0.001). There was no difference in the proportion of correctly assigned scores amongst methods (*P* = 0.612), but there were differences in the relative proportions of under- and over-estimations of body condition (*P* = 0.011). In this respect, the relative proportion of errors that were under- and over-estimates was broadly similar for both the vBCS_measured_ and vBCS_adjusted_ methods, with no significant difference between them (*P* > 0.999, Table 3). However, the overwhelming majority of the errors made with vBCS_subjective_ were under-estimates of body condition (i.e. either obese dogs incorrectly scored as overweight or overweight dogs incorrectly scored as ideal weight; Table 3), significantly more than for both of the other two methods (vBCS_subjective_ vs. vBCS_measured_: *P* = 0.007; vBCS_subjective_ vs. vBCS_adjusted_: *P* = 0.011).

**Table 3:** Results of single-observer validation studies

Method		vBCS_measured_	vBCS_subjective_	vBCS_adjusted_
*Versus BF%* (R_s_)		0.51	0.75	0.65
		*P* < 0.001	*P* < 0.001	*P* < 0.001
*Versus BCS*_*actual*_ (Kappa)		0.51	0.63	0.63
		*P* < 0.001	*P* < 0.001	*P* < 0.001
*Overall scoring accuracy*	Correct	83/125 (66 %)	35/51 (71 %)	90/125 (72 %)
	Under	19/125 (15 %)	13/51 (25 %)	16/125 (13 %)
	Over	23/125 (18 %)	2/51 (4 %)	19/125 (15 %)
*Scoring accuracy (Ideal weight)*	Correct	16/24 (67 %)	13/14 (93 %)	19/24 (79 %)
	Under	n/a	n/a	n/a
	Over	8/24 (33 %)	1/14 (7 %)	5/24 (21 %)
*Scoring accuracy (Overweight)*	Correct	19/37 (51 %)	10/20 (50 %)	19/37 (51 %)
	Under	3/37 (8 %)	9/20 (45 %)	4/37 (11 %)
	Over	15/37 (41 %)	1/20 (5 %)	14/37 (38 %)
*Scoring accuracy (Obese)*	Correct	48/64 (75 %)	13/17 (76 %)	52/64 (81 %)
	Under	16/64 (25 %)	4/17 (24 %)	12/64 (19 %)
	Over	n/a	n/a	n/a

The columns represent data for the three methods for visual body condition scoring (BCS) using photographs. vBCS_measured_: BCS based upon abdominal width to thoracic width ratio measured from a dorsal photograph; vBCS_subjective_: BCS semi-quantitatively assessed from a non-standardised photograph using visual descriptors; vBCS_adjusted_: a refinement of vBCS_measured_, whereby the A:T ratio was first used to estimate BS, but the observer could then modify after examining standardised dorsal and lateral photographs and applying visual BCS descriptors. Rows represent results of performance of each method determined using different parameters. BF% (R_S_): correlation between vBCS method and body fat percentage using Spearman’s rank correlation; BCS_actual_ Kappa: agreement between vBCS method and the actual BCS (9-integer unit system [5] determined by a single observer); Correct BCS assigned: proportion and percentage of dogs correctly scored using each vBCS method and the actual BCS

To examine possible causes for these errors in more detail, the effect of different variables on the proportions of correct and incorrect scores was then assessed. Age (vBCS_measured_*P* = 0.578; vBCS_adjusted_*P* = 0.525; vBCS_subjective_*P* = 0.68), sex (vBCS_measured_*P* = 0.705; vBCS_adjusted_*P* > 0.999; vBCS_subjective_*P* = 0.34), breed type (vBCS_measueed_*P* = 0.528; vBCS_adjusted_*P* = 0.053; vBCS_subjective_*P* = 0.23), coat length (vBCS_measured_*P* > 0.999; vBCS_adjusted_*P* = 0.765; vBCS_subjective_*P* = 0.58), and coat colour (vBCS_measured_*P* = 0.071; vBCS_adjusted_*P* = 0.671; BiCS_subjective_*P* = 0.21) had no effect on the proportions of correctly and incorrectly assigned scores for any of the methods.

The proportions of correct and incorrect estimates of body condition were also stratified into three body condition categories based on the actual body condition score results (Table 3): ideal weight (BCS4-5/9), overweight (BCS6-7/9) and obese (BCS8-9/9). For the vBCS_measured_ method, there was no difference in the proportion of correctly assigned scores amongst dogs in different body condition categories (*P* = 0.150, Table 3). In contrast, significantly more errors were made when assessing the body condition of overweight dogs with the vBCS_adjusted_ method, compared with dogs that were either obese or in ideal weight (*P* = 0.008, Table 3). Most of these errors were overestimates (i.e. overweight dogs being incorrectly scored as obese). When assessing the body condition using the vBCS_subjective_ method, significantly more errors were again made when assessing the body condition of overweight dogs compared with dogs that were obese or in ideal weight (*P* = 0.021, Table 3). However, most of the errors with this method were underestimates (i.e. overweight dogs being incorrectly scored as ideal weight).

### Use of vBCS methods by observers with varying experience

A cohort of 12 observers with a range of levels of experience then tested the three methods of BCS assessment. For all methods, positive associations were observed between body fat mass and the vBCS scores (*P* < 0.001 for all), although the relative strength of the association varied amongst observers (Table 4). The correlation coefficients (R_s_) from the assessments of the experienced observer (AJG) were: 0.74 (vBCS_measured_), 0.84 (vBCS_subjective_) and 0.75 (vBCS_adjusted_). Overall, moderate-to-substantial agreement was seen between actual BCS and the scores determined by all three methods (*P* < 0.001). However, there was variability in the strength of agreement amongst observers, ranging from fair to substantial for vBCS_measured_, from moderate to substantial for vBCS_subjective_, and slight to almost perfect for vBCS_adjusted_ (Table 4). The kappa values of the experienced observer were substantial for vBCS_subjective_ (0.70), and almost perfect for both vBCS_measured_ (0.86) and vBCS_adjusted_ (0.81). The experience of the observer (lay person vs. veterinary trained) did not affect performance of any method (*P* > 0.15 for all).

**Table 4:** Results of multiple-observer validation

Method	vBCS_measured_	vBCS_subjective_	vBCS_adjusted_
*Versus BF%* (R_s_)	0.64 (0.30–0.74)	0.74 (0.65–0.85)	0.70 (0.22–0.80)
	*P* < 0.001	*P* < 0.001	*P* < 0.001
*Versus BCS*_*actual*_ (Kappa)	0.70 (0.32–0.86)	0.55 (0.47–0.70)	0.70 (0.19–0.81)
	*P* < 0.001	*P* < 0.001	*P* < 0.001
*Correct BCS assigned*	30 (19–35)	24 (22–29)	30 (20–33)
	74 % (48–88 %)	60 % (55–72 %)	74 % (50–83 %)

Results are expressed as median (range). The columns represent data for the three methods indirect body condition scoring (BCS) using photographs. vBCS_measured_: BCS based upon abdominal width to thoracic width ratio measured from a dorsal photograph; vBCS_subjective_: BCS semi-quantitatively assessed from a non-standardised photograph using visual descriptors; vBCS_adjusted_: a refinement of vBCS_measured_, whereby the A:T ratio was first used to estimate BS, but the observer could then modify after examining standardised dorsal and lateral photographs and applying visual BCS descriptors. Rows represent results of performance of each method determined using different parameters. BF% (R_S_): correlation between vBCS method and body fat percentage using Spearman’s rank correlation; BCS_actual_ Kappa: agreement between vBCS method and the actual BCS (9-integer unit system [5] determined by consensus between scores of two observers); Correct BCS assigned: proportion and percentage of dogs correctly scored using each vBCS method and the actual BCS

## Discussion

The current study has examined the feasibility of estimating BCS indirectly from photographs using three different methods. Overall, there was moderate agreement between vBCS results and conventional BCS assessed contemporaneously in the same individuals. Further, highly significant correlations were demonstrated between body condition scores from all three vBCS methods and body fat percentage measured by DEXA. The fact that associations between vBCS and DEXA were weaker than between conventional BCS and DEXA is not surprising given the additional information gained from palpation. That said, all conventional BCS assessments were performed by one study observer (SLH) with extensive experience, such that differences in performance (between vBCS and conventional BCS) might partly be related to differences in operator experience. That said, the vBCS results from the study observer conducting the conventional BCS assessment were also inferior. This suggests that, whilst the visual characteristics of body condition in a photograph can provide an estimate of body condition, it cannot substitute for conventional BCS that also includes palpation of the dog.

The three methods performed best when used by an experienced observer, with most dogs being correctly scored. This suggests that, in the right hands, the system has promise as a simple indirect means of gauging body condition. There are various potential applications perhaps the most intriguing of which could be an online tool whereby dog owners would upload photographs for remote reviewing by an experienced observer, with preliminary guidance given to an owner regarding the body condition of their dog. Whilst such an assessment would not replace the use of conventional BCS performed by an experienced operator, it would arguably be better than relying on BCS measurements from owners or other lay people. Owner scores are unreliable demonstrating a systematic tendency to under-estimate condition [7] whether or not training has been provided [8]. If an online tool for assessing vBCS were to be developed, it could be used to advise owners on the likely body condition of their dog, and also to provide cautious guidance as to what action they should take. For instance, owners of dogs that were categorised as ideal weight could be given positive feedback regarding their dog’s status, and advice on what to do to maintain this. In contrast, owners of obese or overweight dogs could be advised to seek veterinary attention. Such a system would have other benefits, for instance, from a research perspective. In this respect, a remote online tool, which removes the subjectivity of an owner, could enable cost-effective collection of large datasets in a simple manner.

The three different vBCS methods were developed after initial validation studies. When two investigators subjectively assessed various photographs, it became evident that some characteristics used to assess body condition in conventional systems were easy to identify in photographs, particularly the abdominal tuck (lateral view) and the waist (dorsal view). We were, therefore, interested in developing both subjective and objective methods of assessing these characteristics. Taking inspiration from human hip to waist ratios [14], initial validation suggested that a ratio of truncal width, measured across the abdomen and across the thorax (A:T ratio), varied amongst dogs in different body conditions. The validation work reported in the current study confirmed these findings, since the A:T ratio correlated moderately well with body fat percentage measured by DEXA. Calculation of this ratio was the basis for the first vBCS method, with cut-points used to categorise dogs into the different body condition categories. In addition to the A:T ratio method, two other vBCS methods were assessed, namely a subjective assessment of photographs taken in a non-standardised view, and combination approach which involved a combination of A:T ratio calculation, and subjective assessment of standardised (dorsal and lateral) photographs. All methods performed relatively well in the hands of an experienced observer, with almost three quarters of images being correctly classified. Whilst not perfect, this does suggest that such a measurement produces a broad guide to body condition, which might be useful clinically.

In the final stage of validation, a group of 12 people, with a range of experience, scored a selection of photographs, and the expectation was that an association with the level of veterinary training would be evident. Thus, it was anticipated that lay observers would perform worst, whilst veterinarians would perform best. In fact, there was no overall difference in the performance of lay and veterinary-trained observers, although marked differences in performance were seen amongst observers. In light of this, further work would be required before recommending widespread use of vBCS methodology. A range of other factors might also influence the visual assessment of body condition, including signalment (most notably breed) and coat characteristics. The latter would be particularly critical since both the colour and the length of the coat might influence how body shape is assessed. Overweight humans commonly use clothing to de-emphasise body shape, for instance by wearing darker colours or clothes with vertical stripes [15]. Interestingly, in the current study no systematic effect of coat colour was seen on the scores returned for any vBCS method. More pertinently, coat length could certainly influence the use of a visual scoring system, since there would be a tendency for the exact contours of the body to be obscured. Surprisingly, However, we cannot discount an influence of coat length entirely given the small study population, and further work would be recommended.

One factor that did influence the accuracy of BCS determination from photographs was the actual body condition score of the dog, correct scores more likely when dogs were in ideal weight or obese than for dogs that were overweight. For the vBCS_adjusted_ method, most of the errors made were in incorrectly classifying obese dogs as overweight, with fewer errors made in distinguishing ideal weight from overweight dogs. In contrast, for the vBCS_subjective_ method, whilst it was uncommon for ideal weight dogs to be incorrectly classified as overweight, most errors made in scoring overweight dogs were to under-estimate their actual body condition (i.e. incorrectly classify them as ideal weight). Any future use of a photographic BCS approach should consider these findings. For example, if epidemiological studies used such methods, the true prevalence of overweight status within the population studied could be under-estimated.

The study has a number of limitations that must be considered. Firstly, all images were reviewed retrospectively and had not been taken with the specific purpose of validating a photographic BCS. This might have reduced the options available when deciding upon a method of assessment of body condition. The use of a database of retrospectively collected images might also have affected the performance of the system, and it is possible that a scoring system would perform differently (possibly better) were photographs to be taken specifically for the purpose.

A second study limitation relates to the population used for the study, namely a small cohort of overweight or obese dogs, with photographs having been taken either before or after weight loss. Given the small study size, some of the statistical analyses might have been underpowered. Further, body shape of dogs after weight loss might not be representative of dogs in ideal condition that had never been overweight. Moreover, images were not included of underweight dogs, to determine whether or not the same system could be used to determine condition of dogs in suboptimal condition. That said, the performance of the methods would arguably have been better were a broader range of body conditions to have been assessed. Therefore, it is recommended that further studies now be performed to assess a larger population of dogs, with a wider range of body condition scores and including underweight dogs and those who have remained in ideal condition for their whole life.

A third limitation was the fact that many of the standardised and non-standardised images were taken from the same dogs and, given that observers scored both sets, there was a possibility that observers could recognise the same dogs and their score might be influenced. To compensate for this, the order in which the images were viewed was randomised, with half of the observers viewing the standardised images first, and the others viewing the non-standardised image first. Related to this issue was the fact that the dogs attended a weight management clinic run by two study authors that participated as observers (AJG, SLH), and it is possible that prior knowledge of body condition might have influenced their scores. That said, whilst BCS is recorded at the beginning and end of each weight loss programme, it is not used for clinical assessment. Instead, body composition results from DEXA are used to determine the degree of adiposity, to calculate target weight, and to determine the point at which optimal body composition is attained.

A final limitation is that there might have been differences in the approach used by different observers when performing measurements, for if they were to take measurements at different sites. For the future, consistency could be improved by ensuring a standard image size is used, for instance where head-to-tail-base length base of tail length is a standard 18 cm (red line). Chest and waist measurements could then be made at standard distances e.g. 5 cm and 10 cm along the midline from the nose. However, whilst more automated, given differences in breed conformation, it is likely that these points would represent different anatomical sites, so issues with reliability might not be completely circumvented.

## Conclusions

The current study has reported initial validation studies for determining body condition from photographs. When performed by an experienced observer, all methods correlated positively with body fat percentage measured by DEXA, although performance was inferior to conventional BCS assessment. Further, more variability was seen when observers with a range of experience used the same methods. Therefore, whilst photographic methods performed by an experienced observer may be valid for initial clinical screening and also in population studies, conventional BCS assessment remains the best technique for confirming the body condition of an individual. Further studies are now warranted in order to refine methods of assessment and to determine the utility of such an approach as an initial screening tool.
